# The Influence of Reaction Conditions on the Properties of Graphene Oxide

**DOI:** 10.3390/nano14030281

**Published:** 2024-01-30

**Authors:** Miroslav Huskić, Dejan Kepić, Duška Kleut, Miran Mozetič, Alenka Vesel, Alojz Anžlovar, Danica Bajuk Bogdanović, Svetlana Jovanović

**Affiliations:** 1 Faculty of Polymer Technology, 2380 Slovenj Gradec, Slovenia; 2 Vinča Institute of Nuclear Sciences, National Institute of the Republic of Serbia, University of Belgrade, 11000 Belgrade, Serbiasvetlanajovanovic@vin.bg.ac.rs (S.J.); 3 Jožef Stefan Institute, 1000 Ljubljana, Slovenia; miran.mozetic@ijs.si (M.M.); alenka.vesel@ijs.si (A.V.); 4 National Institute of Chemistry, 1000 Ljubljana, Slovenia; alojz.anzlovar@ki.si; 5 Faculty of Physical Chemistry, University of Belgrade, 11158 Belgrade, Serbia; danabb@ffh.bg.ac.rs

**Keywords:** graphene oxide, synthesis, thermal characterization, Raman spectroscopy, XPS, DLS, TGA, UV-VIS, XRD

## Abstract

The present study focuses on correlations between three parameters: (1) graphite particle size, (2) the ratio of graphite to oxidizing agent (KMnO_4_), and (3) the ratio of graphite to acid (H_2_SO_4_ and H_3_PO_4_), with the reaction yield, structure, and properties of graphene oxide (GO). The correlations are a challenge, as these three parameters can hardly be separated from each other due to the variations in the viscosity of the system. The larger the graphite particles, the higher the viscosity of GO. Decreasing the ratio of graphite to KMnO_4_ from 1:4 to 1:6 generally leads to a higher degree of oxidation and a higher reaction yield. However, the differences are very small. Increasing the graphite-to-acid-volume ratio from 1 g/60 mL to 1 g/80 mL, except for the smallest particles, reduced the degree of oxidation and slightly reduced the reaction yield. However, the reaction yield mainly depends on the extent of purification of GO by water, not on the reaction conditions. The large differences in the thermal decomposition of GO are mainly due to the bulk particle size and less to other parameters.

## 1. Introduction

Graphene is a one-atom-thick sheet of hexagonally packed carbon atoms that exhibits several interesting properties, such as a high specific surface area (2630 m^2^/g), high elastic modulus (1 TPa), and excellent electrical (6000 S/cm) and thermal conductivity (3000–5000 W/mK) [1,2]. These properties make graphene a good candidate as a nanofiller for the fabrication of high-strength, high-thermal, and electrically conductive polymer nanocomposites. There are several direct methods to produce graphene, such as mechanical cleavage of graphite, chemical vapor deposition, epitaxial growth [1,2], unzipping of carbon nanotubes [3], and electrochemical exfoliation [4]. However, these methods are not suitable for mass production, which is required for the manufacturing of conductive polymer composites. Larger quantities of graphene can be produced by the chemical or thermal reduction of graphene oxide (GO). GO is not only an interesting material as a precursor for graphene but can also be used as a reinforcing filler in polymer nanocomposites. GO has therefore been the subject of intensive research for several years [5,6,7,8,9].

The exact structure of GO is still not clear, but we do know that there are hydroxyl, epoxy, carbonyl, and carboxyl groups bound to graphene sheets [1,6,10]. However, it is worth noting that there should be a distinction between graphene oxide and graphite oxide (GrO). During the oxidation of graphite, GO is formed since it is dispersed in acid on a molecular level. This might be argued due to the layered structure of the slurry, as will be shown later. However, the layered structure is a consequence of intercalated water (or the other polar solvent) between the GO sheets. Adding more water to the slurry will increase the interlayer thickness and finally exfoliate the GO sheets. On the other hand, once it is dried, the sheets stick together, and GrO is formed. The difference between GO and GrO is sometimes attributed to the number of layers (<10 is GO; >10 is GrO) [6]. GrO can be dispersed in water but not fully dissolved. In our experience, GrO precipitates from water dispersion within a few days. Therefore, the majority of characterizations of GO found in the literature are based on GrO.

GO is relatively easily synthesized by the oxidation of graphite using various methods like Hummers’, Brodie, and Staudenmaier, which are being modified and improved [11,12]. Additionally, GO has also been synthesized mechanochemically [13] and electrochemically [14].

There are several review articles covering the chemical synthesis and modifications of GO [6,8,15,16,17]. However, it is very often hard to compare the results of different authors who use almost identical materials. The reason is that very different ratios of graphite to oxidant and acid were used, at various reaction times, at room or increased temperatures. Sometimes even ultrasound was used to promote the reaction [18]. Various methods might produce GO with different chemical structures, which results in different properties and reactivity. For example, it has been observed that GO produced using Hummers’ method has lower reactivity than GO produced using Brodie’s method [19].

The present study is focused on the influence of three parameters, namely graphite particle size, graphite-to-oxidant (KMnO_4_) ratio, and graphite-to-acid ratio, on the structure and properties of GO prepared using the so-called modified Hummers’ method or Tour’s method [12]. The influence of graphite particle size on GO particle size has already been investigated, but the results reported by different groups vary considerably and are therefore not conclusive. For example, Zhou and Liu produced GO particles up to 200 μm in size and reported that the size of GO is somehow smaller but comparable in size to the parent graphite particles [20]. Chen et al. used sieved graphite and obtained larger GO particles with larger graphite particles. However, these authors used various reaction conditions (time of reaction and graphite/acid ratio) for the various graphite sizes [21]. Other authors claim that the use of large-size natural graphite flakes as precursors does not necessarily produce large GO sheets [22].

The literature survey revealed that various ratios of graphite to KMnO_4_ were used to synthesize GO; however, no systematic study of the influence of the ratio on the final properties of GO was performed, so the exact correlation is yet to be reported.

This study comprehensively considers three different synthetic factors and addresses their effects on GO yield and its structural properties. Thanks to the multifactorial approach, it brings new data regarding the effect of the viscosity of the reaction mixtures and critically discusses the issues in the synthetic and thermal characterization problems in GO. Several other problems associated with GO synthesis and characterization will be disclosed and discussed in this article, and the differences will be explained.

## 2. Materials and Methods

### 2.1. Materials

Expanded graphite Sigratherm^®^ GFG 130 (average particle size: 130 μm) is the product of SGL Carbon GmbH, Meitingen, Germany. It was fractionated into three phases by sifting it through 45-µm and 100-µm sieves. The graphite flakes obtained by sifting through a 45-μm sieve were designated as G45; flakes that remained on a 45-µm sieve and passed a 100-µm sieve were designated as G45+; and flakes that did not pass a 100-µm sieve were designated as G100+.

H_2_SO_4_ (95–97%), H_3_PO_4_ (85%), H_2_O_2_ (30%), KMnO_4_, and methanol (MeOH) were purchased from Merck KGaA, Darmstadt, Germany and used as received.

### 2.2. Synthesis of Graphene Oxide (GO)

GO was prepared according to the modified Hummers’ or Tour’s method [12]. A total of 1 g of graphite flakes was added to a 9:1 mixture of concentrated H_2_SO_4_/H_3_PO_4_ acids (60 mL or 80 mL) in a 250-mL beaker equipped with an overhead polytetrafluoroethylene (PTFE) stirrer. KMnO_4_ (4 g or 6 g) was added in a 1 g portion per hour, and the reaction proceeded at room temperature for 20 h. At the end of the reaction, ice (≈0.2 kg) was added, followed by 5 mL of 30% H_2_O_2_. The product was divided into six centrifuge tubes (50 mL), centrifuged at 9000 rpm for 15 min, and decanted. The product was successively washed with water, 10% HCl (twice), and MeOH (three times). This procedure was used to obtain as much pristine GO as possible since it was found that purification with water leads to a change in GO structure [10]. Each time, the centrifuge tube was filled up to 48 mL, mixed thoroughly to redisperse the particles, and centrifuged/decanted again. The purified GO samples were either dried or kept as MeOH dispersion (≈8 wt.% of GO) and stored in a fridge. The GO samples were designated according to their graphite size (i.e., GO45, GO45+, and GO100+) and graphite:KMnO_4_:acid ratio (for example, GO45 1:4:60).

### 2.3. Characterization

The size of the graphene oxide particles in deionized (DI) H_2_O was measured by dynamic light scattering (DLS) using a Malvern Zetasizer Nano-ZS. Scattering was measured at an angle of 173°. The concentration of GO in the deionized water was 0.05 mg/mL. The GO particle size of the dried as well as slurry samples was determined. The carbon black and H_2_O refractive indices of 1.470 and 1.3317, respectively, were used in calculations.

The morphologies of these samples were evaluated through atomic force microscopy (AFM) on a Quesant microscope (Agoura Hills, CA, USA). operating in tapping mode in the air at room temperature. The samples were dispersed in water, drop cast on a Si substrate, and imaged after drying. Silicon tips (Nano and More, Gmbh, Wetzlar, Germany) with a constant force of 40 N/m were used for AFM imaging. The images were analyzed using Gwyddion software (version 2.44).

The chemical composition of the GO particles was investigated by X-ray photoelectron spectroscopy (XPS) using a model TFA XPS from Physical Electronics (Chanhassen, MN, USA). The GO samples were excited with monochromatic Al Kα1,2 radiation at 1486.6 eV. Survey-scan spectra were measured at a pass energy of 187 eV and an energy step of 0.4 eV. High-resolution spectra of carbon were measured at a pass energy of 29.35 eV and an energy step of 0.125 eV. The measured spectra were analyzed using MultiPak v8.1c software supplied by the XPS manufacturer. Carbon C1s spectra were fitted using a Gaussian–Lorentzian function and Shirley background subtraction.

The thermal stability of the GO samples was determined using a Mettler Toledo (Greifensee, Switzerland) TGA/DSC 1 instrument. The samples of »3 mg were heated from 40 °C to 800 °C at a heating rate of 5 K/min in a nitrogen flow rate of 20 mL/min.

The characterization of the GO particles by X-ray diffraction (XRD) was performed with the X-ray powder diffractometer PANalytical X’Pert PRO MPD (CuKα_1_ radiation = 1.5406 Å) in 0.033° steps from 2θ = 2.0° to 60°. The sample of GO slurry in methanol was covered with a Kapton foil to prevent the evaporation of the methanol. Other samples were vacuum-dried at 40 °C for 24 h prior to XRD characterization. The basal spacings were calculated according to Bragg’s law.

Raman spectra were recorded on a DXR Raman microscope (Thermo Fisher Scientific, Waltham, MA, USA). Each spectrum was obtained at room temperature by using a 532-nm excitation line with a power of 5 mW. The spectral resolution was 1 cm^−1^, and the acquisition time was 10 × 10 s.

The UV-VIS absorption spectra were recorded with a LLG-uniSPEC 2 spectrophotometer. The samples dispersed in water were recorded in quartz cuvettes at room temperature.

The structural properties of GO were investigated using ATR-FTIR spectroscopy. The measurements were performed on a Thermo Scientific Nicolet iS20 (Waltham, MA, USA) FTIR spectrometer equipped with a single reflection diamond crystal. All measurements were conducted at room temperature. The FTIR spectra of different GO samples were recorded in the range of 400 to 4000 cm^−1^ at 64 scans per spectrum with a resolution of 2 cm^−1^.

Water contact angle (WCA) measurements were carried out by using the sessile drop method on the Theta Lite contact angle meter (Biolin Scientific, Stockholm, Sweden). To obtain thin films of GO samples suitable for measuring the WCA, 100 µL of the GO sample dispersed in water was drop cast on freshly cleaned microscope slides and dried at 60 °C. This procedure was repeated several times until homogeneous films with a rather smooth surface were prepared. To acquire the WCA, a 6-μL droplet of deionized water was carefully placed on the samples using a microsyringe. All WCA measurements were performed at ambient conditions (25 °C). The data were analyzed using OneAttension software (version 4.0.3).

## 3. Results and Discussion

One of the first observations during this work was that not only the ratio of reactants is important but also the amount of reagents. In the initial phase of the synthesis of graphene oxide particles, the graphite flakes became more hydrophilic due to the oxidation in the mixture of acids and potassium permanganate, resulting in swelling and increased viscosity. The larger the graphite particles, the more viscous the paste. In our previous experiments, 10 g of graphite (GFG 130) was oxidized in 500 mL of the acid mixture (ratio: 1:50) [9]. Mixing was not very effective, as the reaction mixture was very viscous at the end of the reaction. However, using only 1 g of graphite and a smaller beaker, as in the current experiments, it was not possible to carry out a reaction using the same graphite-to-acid ratio (1:50). As soon as the viscosity increased, the product stuck to the glass wall and the stirrer only mixed the air. Therefore, the graphite: acid ratio was increased to 1:60 and 1:80 in these experiments.

### 3.1. Determination of Reaction Yield

Since the exact chemical structure of GO is not known, the reaction yield is usually determined as the weight of GO divided by the weight of the graphite used in the reaction. The quantitative separation of GO from the reaction mixture is necessary to determine the reaction yield. There are two methods frequently reported for the separation and purification of GO, namely centrifugation and filtration. However, these methods are usually inadequately described in the literature. For example, Rourke et al. used a modified Hummers’ method and purified GO by centrifugation, discarding the supernatant, and resuspending the residue at least ten times [23]. Unfortunately, Rourke et al. did not specify which liquid was used for their purification. If water was used ten times, the yield of GO would have been very low, as some GO is always soluble in water and lost.

Chen et al. purified GO by dialysis, followed by centrifugation. The yield they obtained was 92–96% [11]. A similar yield (»100%) was obtained by Daud et al. [24], and 110% by Bera et al. [18] A very low yield (30%) was obtained by Xu et al. However, unlike the others, Xu et al. discarded the sediment after centrifugation and only used the soluble fraction of GO particles [25].

Hussein et al. prepared GO according to the Staudenmaier method and washed it first with 5% HCl, then with water, and the slurry was vacuum-filtered [26]. This seems simple, but, according to our experience, the filtration should be explained in detail, especially what kind of filter was used. The filtering of GO/water is not trivial, and the filtering time is also an important parameter. In our experiments, we tried several filter papers and glass filters with very little success; only very small amounts of GO can be filtered before the pores become clogged. The best filter seems to be the Isopore^TM^ polycarbonate membrane, which enables the production of thin films; however, filtering larger quantities is also very problematic (we only tested membranes with 0.22 μm pore size and 47 mm in diameter).

Increasing the ratio of graphite to KMnO_4_ from 1:4 to 1:6 increased the reaction yield, as shown in Table 1. However, the effect is small, even for the smallest particles (GO45). Obviously, the 1:4 ratio enables enough oxidant for almost optimal yield. This is consistent with previous findings [10].

Table 1 also shows the yield for the cases in which the GO synthesis procedure was performed using two different acid amounts (60 mL and 80 mL). The influence of the ratio of acid to graphite on the yield is not straightforward. For the smallest particles, a certain increase in yield was observed with increasing ratio, while a decrease was observed for larger graphite particles. Due to the lower concentration of KMnO_4_ at a higher ratio, which could decrease the reaction rate, the decrease in yield was somehow expected. The unexpected increase for the smallest particles can be related to the higher mobility of the reagents in a much less viscous solution. The viscosity of the GO slurry at the end of the synthesis increased with increasing graphite particle size. All the products prepared in 60 mL of acid were pasty at the end of the reaction. The products prepared from G45 in 80 mL were viscous liquids, while the products prepared from G45+ or G100+ were still pasty. Therefore, the mobility of the reagents only strongly increased for G45, which might lead to a slightly increased yield.

However, all these differences in yields might be a consequence of the different solubilities of the products due to different chemical structures and different particle sizes, as well as due to small and unintended differences during the purification process. We observed that a part of GO is always soluble in water and forms a saturated solution. The rest of GO is dispersed and can be separated by centrifugation. Therefore, our results summarized in Table 1 do not depend so much on the reaction conditions (ratio of reactants) but rather on the purification method. If more water is used in the purification, a lower yield is observed.

To prove this, we took a large batch of GO/methanol slurry from previous studies and performed a purification with demineralized water and centrifugation. Approximately 2.5 g of slurry (11% GO in methanol) was weighed into three centrifuge tubes, and approximately 35 g of water was added. The centrifuging time was 2 h at 9000 rpm. The soluble part was discarded, and water (approximately 25 g, as the swelling of GO in water is greater than in methanol) was added to tubes #2 and #3, mixed thoroughly, and centrifuged again. This process was repeated once more for tube #3. All the samples were dried by lyophilization for 48 h and then in vacuo at 40 °C for 8 h. The loss of GO was 15%, 25%, and 58% after the first, second, and third purification steps, respectively.

### 3.2. XPS Analysis of GO

**Figure 1:**
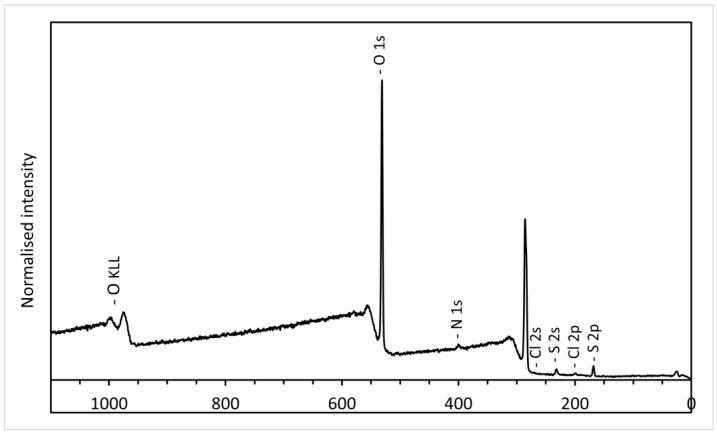
The XPS survey spectra of GO100+ 1:6:80.

The reaction yield in our experiments is very high compared to the previously published data. This is a consequence of the fact that we only performed one wash using water. Even when 10% HCl is used for washing, GO is much less soluble, less swellable, and easier to separate by centrifugation. Although it is desirable to have a good yield, this raises the question of the purity of GO purified by our method. Figure 1 shows the XPS survey spectra of GO100+ 1:6:80 as an example.

Only the peaks of carbon and oxygen are clearly visible, which indicates that the concentration of impurities, except for sulfur and chlorine, is below the detection limit. Therefore, the XPS results show that the GO we prepared is just as pure as reported by others. Obviously, the acids and salts were soluble in 10% HCl and/or alcohol, and the rest of the acids were mostly removed using methanol. The presence of chlorine (0.2–0.3 at.%) and sulfur (1.5–2.0 at.%) was confirmed, and similar quantities were frequently mentioned in other publications about the synthesis of GO [10,21,27].

**Figure 2:**
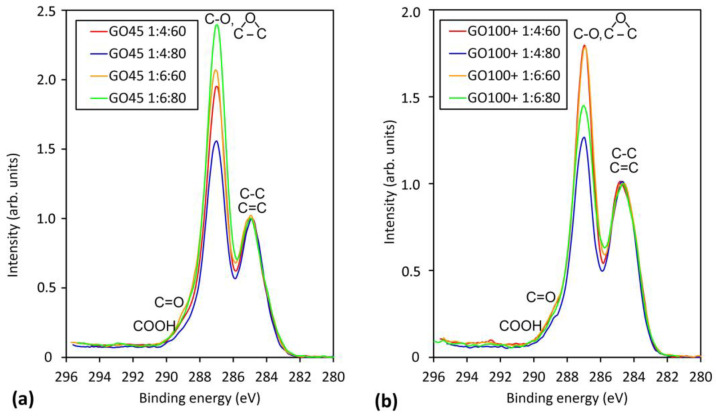
The high-resolution C1s spectra of (**a**) small particles, GO45, and (**b**) large particles, GO100+.

Due to a relatively small difference in the chemical structure of GO, the XPS analysis was only performed for the GO synthesized from the smallest and largest graphite particles. The high-resolution C1s spectra of these samples are shown in Figure 2 and explained later in the text. Table 2 represents the results of the proportions of various bonds obtained by fitting the high-resolution C1s spectra (Figure S1 in the Supplementary Materials). The C1s spectra were fitted using four components corresponding to C-C and C=C (sp^2^), C-O (epoxy or hydroxyl), C=O, and COOH. The majority of oxygen is found in the epoxy or hydroxyl groups.

#### 3.2.1. The Influence of the Graphite Particle Size

According to the O/C ratios in Table 2, the smaller particles were more oxidized than the larger ones. The one exception is GO45 1:4:60, which seems to be oxidized to a comparable level as the large particles. The average percentage of C-C bonds was 39.0% and 44.7% for GO45 and GO100+, respectively. The average oxygen-to-carbon ratio (O/C) was also higher for GO45 (0.58) than for GO100+ (0.51). According to these results, the reaction yield should be higher with the smaller particles (because of their higher oxygen content, and thus the mass should be greater), but this was not the case (Table 1). It seems that the smaller particles were more soluble in water due to a higher degree of oxidation and smaller size, so they were lost during the purification process. This may explain a deviation in GO45 1:4:60. Small and strongly oxidized particles could have been further broken down by mixing a viscous solution, became more soluble, and were removed by washing.

The concentration of carbonyl bonds is higher for the smaller particles, which is in agreement with previous results [9]. The carbonyl bonds are formed at the edge atoms and on the edges of vacancy defects in the basal plane. Since the smaller particles exhibit a larger ratio between the edge and plane carbon atoms than the larger ones, a larger number of the carbonyl groups is expected for the smaller particles. The edge atoms of graphene oxide are also terminated by carboxyl (COOH) groups. The concentration of these groups is approximately 4% for both types of GO material. Here, it has to be stressed that the C=O and COOH groups partially overlap in the C1s spectra, so the exact concentration of the carboxyl group is difficult to determine. Increasing the acid-to-graphite ratio from 60 to 80 increased the concentration of C=O and COOH for GO45, while the opposite effect was observed for GO100+.

The C-O bonds can be either in the epoxy or hydroxyl functional groups, but unfortunately, the XPS technique does not distinguish between these groups since the differences in the chemical shift are marginal.

#### 3.2.2. The Influence of the Ratio of Reactants

Increasing the ratio of graphite to oxidant (KMnO_4_) from 1:4 to 1:6 decreased the amount of C-C/C=C bonds, indicating more efficient oxidation, but the differences are rather small. The amount of C=O and COOH increases with increasing oxidizing agent and acid content for the small particles, while the opposite trend is observed for the large particles.

### 3.3. Characterization of GO Using UV-VIS, FTIR, and Raman Spectroscopy

**Figure 3:**
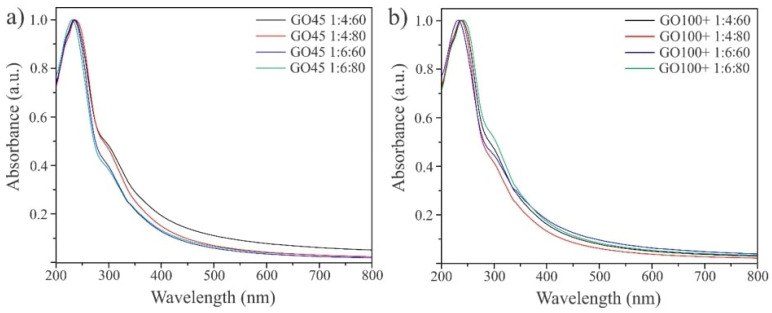
The UV-VIS spectra of the obtained GO samples: samples of GO produced from GO45 (**a**) and GO samples produced starting from GO100+ (**b**).

The structure of the obtained materials was analyzed using UV-VIS, FTIR, and Raman spectroscopy. The UV-VIS absorption spectra (Figure 3) have one dominant peak at ~235 nm and a shoulder at ~300 nm, confirming that all the samples have similar structures. The peak at 235 nm originates from the π-π* transition of the aromatic C=C bonds, while the shoulder at ~300 nm originates from the n-π* transition of the C=O bonds [12]. The shoulder is present in all GO45 and GO100+ samples and indicates the presence of oxygen functional groups in the structure of oxidized graphene, which is in good agreement with XPS analysis.

**Figure 4:**
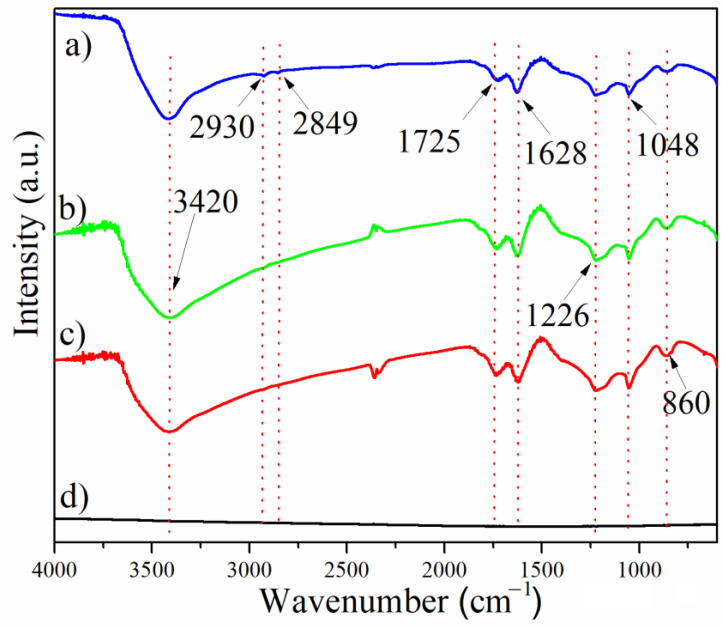
The FTIR spectra of GO samples: GO45 1:4:60 (**a**), GO45 1:6:60 (**b**), GO100+ 1:4:60 (**c**), and graphite powder (**d**).

The presence of functional groups in the GO samples was analyzed using FTIR spectroscopy, and the results are shown in Figure 4. All GO samples (GO45 1:40:60 (a), GO45 1:6:60 (b), and GO100+ 1:4:80 (c)) showed the same bands in their FTIR spectra, while no band was detected in graphite (Figure 4d). The band assigned to the stretching vibrations of the carbonyl groups was observed at 1725 cm^−1^. The band at 1626 cm^−1^ originates from the vibrations of the C=C bonds. The bands at 3420 cm^−1^ and 1048 cm^−1^ originate from C-O-C and the stretching and bending vibrations of the OH groups. Weak bands at 2930 cm^−1^ and 2849 cm^−1^ are the result of stretching vibrations of the H-C bonds, and the band at 860 cm^−1^ is associated with the vibrations of the epoxide groups. These analyses demonstrate the successful chemical oxidation of graphite and the binding of oxygen-containing functional groups.

**Figure 5:**
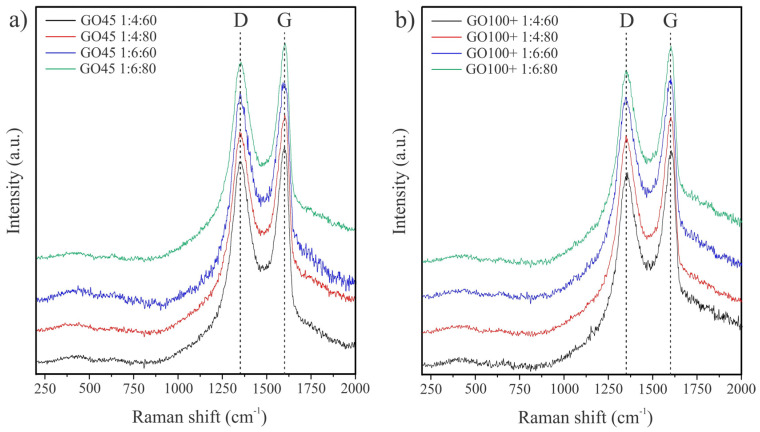
The Raman spectra of synthesized GO samples: GO produced starting from GO45 (**a**) and GO obtained from GO100+ (**b**). The spectra are displaced vertically for clarity.

The Raman spectra of GO (Figure 5) are composed of two dominant features, the G and the D peaks, which appear at ~1580 and ~1350 cm^−1^, respectively [28]. The G peak is attributed to the vibration of sp^2^-bonded carbon atoms in a two-dimensional hexagonal lattice and is the characteristic Raman peak for all carbonaceous materials arranged in hexagons. On the other hand, the D peak is either due to the structural defects [29,30] or the edges of the nanosheets [31]. The intensity ratio of these two bands (*I_D_/I_G_* ratio) can serve as an indicative parameter to estimate the quality of graphene-like materials [30]. The values of the *I_D_/I_G_* ratio between 0.88 and 0.95 indicated defects in the sp^2^ carbon structure of graphene. All GO samples have comparable *I_D_/I_G_* ratio values (Table 3). However, slightly lower values have been calculated for the GO obtained from GO100+, which is a result of lower oxidation.

### 3.4. Hydrophilicity of GO

Due to the presence of oxygen functional groups, the thin films prepared from GO samples exhibit good hydrophilicity (Table 4). The highest water contact angle of 34.3° is measured for the GO100+ 1:4:80 sample. Taking into account the data in Table 2, this is expected, as this sample has the lowest O/C ratio. The variations in the WCA for the other samples could be related to the lack of uniformity of the films, which is influenced by the deposition method [32,33], or by variations in their oxidation state. The epoxy and C=O groups contribute to oxidation but are not as polar as the hydroxy and -COOH groups.

### 3.5. Particle Size Determination

The particle size of GO has frequently been determined by DLS, although this technique was developed for spherical particles and not planar particles [18,34,35]. Since the GO particles are planar, the results obtained show a cumulative effect of the size distribution of the nanosheets, their position with respect to the direction of a light beam, and the solvation and crumpling of the 2D nanosheets [18]. The average particle size of planar particles determined by DLS is usually several times smaller than the average lateral size of the same particles determined using TEM or SEM [9]. However, for less than micron-sized nanoplates, the empirical correlation between the TEM and DLS results was developed based on the measurements of different exfoliated nanosheets, including GO [34].

As explained above, the size of the GO particles generally depends on the size of the graphite precursor, but this is not always necessary. However, GO particles are usually much smaller (1–10 μm) than the size of the graphite particles (a few μm and up to a few mm), and the observed differences in GO particle size synthesized from small and large graphite particles are usually small [21,22].

**Figure 6:**
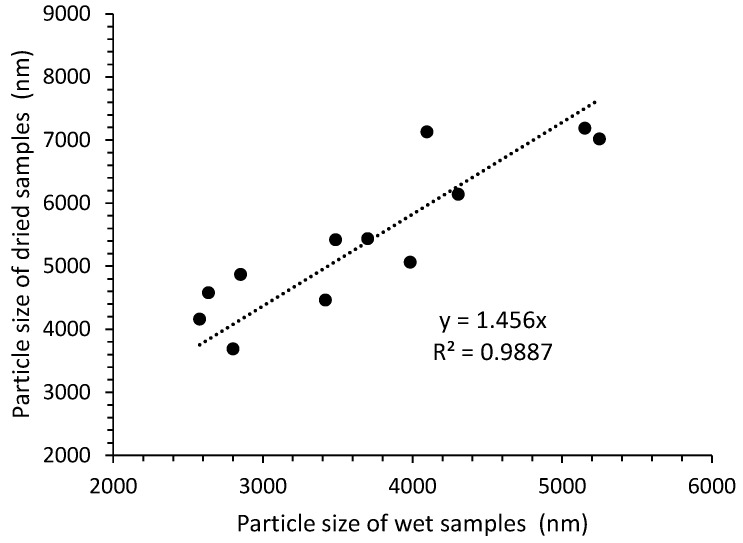
The correlation between the size of dried GO particle size and the size obtained from the slurry.

The DLS measurements were performed for both the dry and wet samples, i.e., taken directly from the methanol slurry. However, the concentration of GO was the same for all the samples. The results in Table 5 show that the size of the dried GO particles is always, on average, almost 50% larger than the size of the as-synthesized particles (Figure 6). This might be explained by strong interlayer binding forces that hinder the complete exfoliation of the stacked graphite oxide back into graphene oxide sheets or, even more likely, by chemical reactions between the sheets that crosslink them. The latter explanation can be supported by the fact that the solubility of GO decreases over time and, in our experience, after a few years, only a slightly swelled slurry is mostly obtained even in water.

The results presented in Table 5 corroborate the findings of Jia et al. in that the use of large-sized graphite flakes as precursors does not necessarily lead to large GO sheets and that the processing conditions have to be optimized for the synthesis of large particles [22]. The influence of the reaction conditions on the particle size is inconclusive. The results scatter between 2.5 μm and 5.2 μm for the slurry samples, and 4.2 μm and 7.2 μm for the dried samples.

One possible explanation for the scattering of these results is the fine interplay between the viscosity of the system, rate of oxidation, and mechanical mixing. The observations derived from our experiments can be summarized as follows:

- Graphite-to-oxidant ratio: Adding more oxidant should result in more oxidized GO. The graphite flakes should be more damaged and, therefore, smaller. However, this effect was only observed for the smallest particles (GO45).
- Acid-to-graphite ratio: Acid enables oxidation and acts as a solvent with a diluting effect. Increasing the ratio from 1:60 to 1:80 reduces the concentration of reactants, which generally reduces the reaction rate. This should result in less oxidation (at a constant reaction time) and a larger particle size. Additionally, the viscosity of the reactant mixture is reduced by the addition of more acid, which reduces the mechanical breaking of the particles.
- Graphite particle size: The smaller the particle size, the higher the proportion of end-C atoms, which are more easily accessible and more reactive than inner C atoms. The oxidation of larger graphite particles is, therefore, slower, which leads to a larger particle size. The viscosity of the reaction mixture, after the reaction has started and GO has formed, increases with increasing graphite particle size. Increased viscosity reduces the mobility of reactants, which could reduce the reaction rate. Therefore, larger GO particles are expected to be obtained by using larger graphite particles. However, the reaction mixture must be mixed to ensure the homogeneous oxidation of all graphite particles. Mechanical mixing of very viscous slurry inevitably leads to the breakage of the particle and, thus, a size reduction. The extent of the breakage depends on the viscosity, which changes with the degree of oxidation and GO concentration.

Therefore, it is almost impossible to produce GO particles in a batch under the same conditions as the viscosity of the reaction mixture depends on the size of the graphite particles. It is only possible to approach the same conditions if the reaction is carried out in a very large amount of acids, which is both economically and ecologically unacceptable.

**Figure 7:**
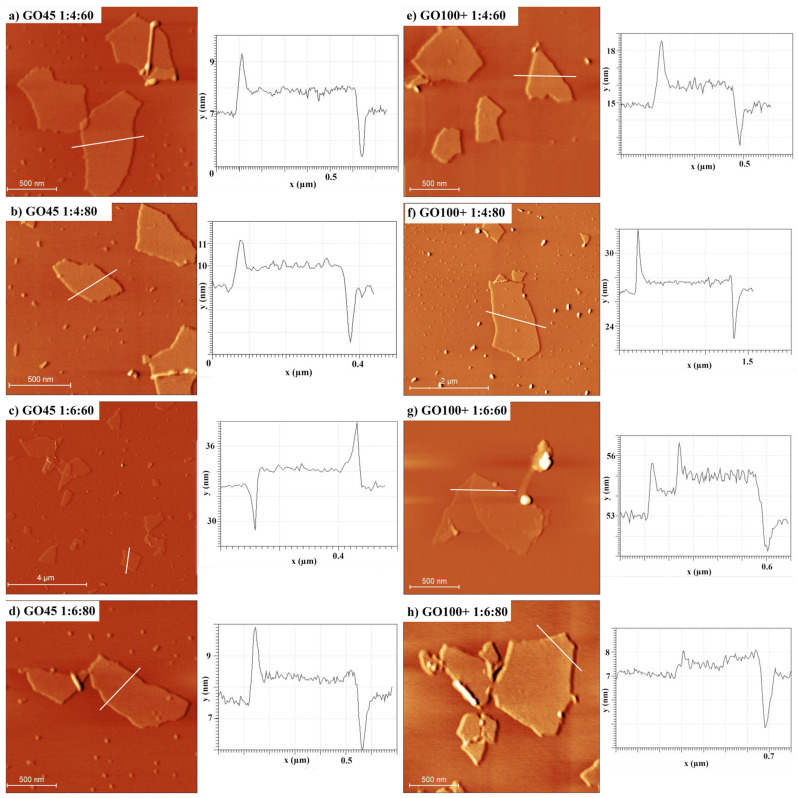
The AFM images of the obtained GO samples along with their profiles.

The AFM analysis was performed to gain a better insight into the morphologies of the prepared GO samples. The AFM images (Figure 7) of GO on an atomically flat surface (Si) show a broad distribution of GO sheet size from several nanometers up to several micrometers. GO sheets are generally flat, and only a small portion of wrinkles and overlaps are visible on the AFM images. The average thickness of GO sheets is around 1 nm. It has been reported that the presence of epoxy and hydroxyl groups in graphene increases the thickness of the graphene layer by about 0.4 nm [36], so the measured profiles indicate the presence of predominately single-layer GO sheets.

### 3.6. XRD Analysis of GO

**Figure 8:**
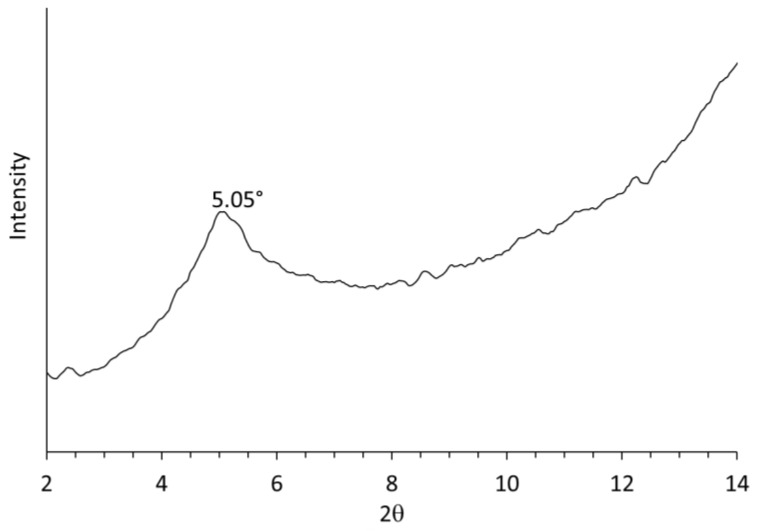
The XRD diffractogram of the GO slurry in methanol.

The XRD peak position in GO, which determines the basal spacing (d_002_), is mainly influenced by the degree of oxidation and humidity. The basal spacing of anhydrous GO was determined to be 0.6 nm [37]. Due to the lyophilic character of GO, water or polar solvents such as methanol can penetrate the interlayer space and increase the basal spacing. The XRD diffractogram of the GO slurry with about 8 wt.% GO in methanol is shown as an example in Figure 8. The d-spacing, as calculated using Bragg’s equation, was 1.75 nm.

**Figure 9:**
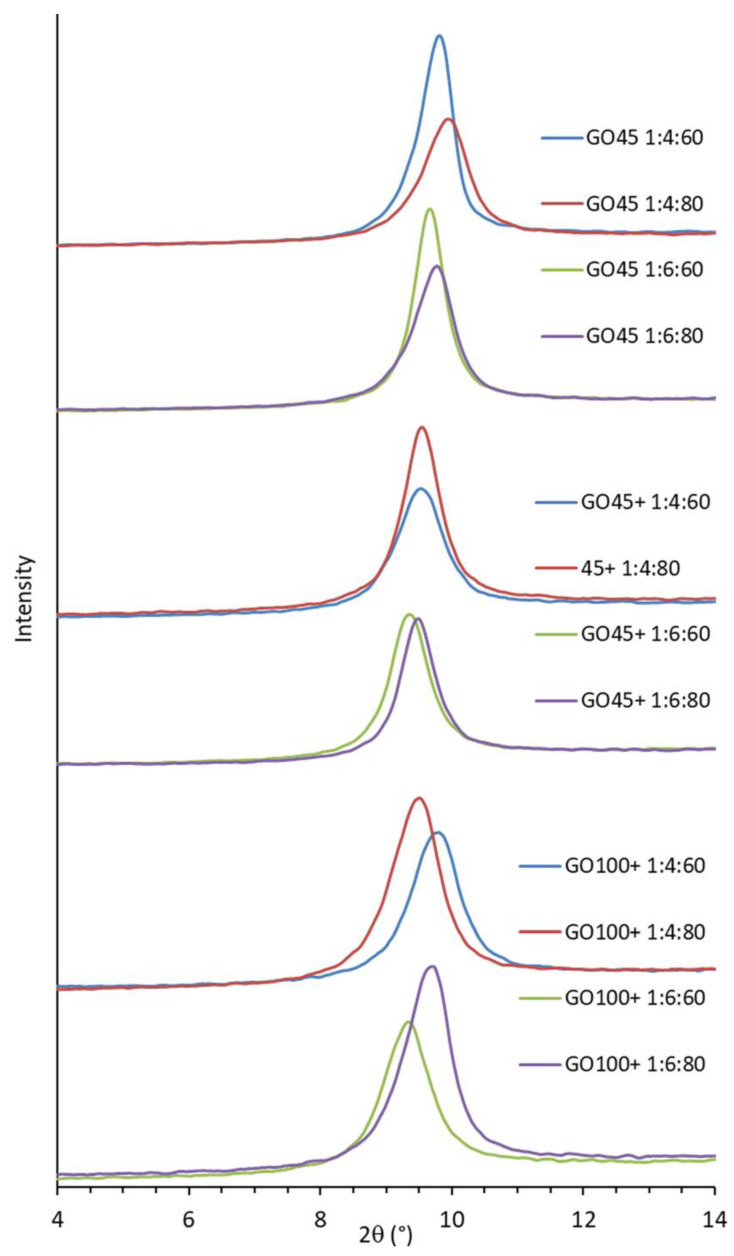
XRD diffractograms of GO synthesized under various conditions.

To obtain XRD data independent of moisture or methanol content, all samples were vacuum-dried at 40 °C for 24 h before measurement. The shape and position of the XRD diffractions depend on the reaction conditions (Figure 9). The d-spacing and crystallite size were calculated using Bragg’s (2d_hkl_sinθ = nλ) and Scherrer’s (B(2θ) = Kλ/L⋅cosθ) equations, respectively (Table 6).

Some results are not very conclusive when GOs are compared by graphite size, but trends can be observed within each sample group.

The d-spacing decreased with increasing acid concentration for the smaller particles (GO45 and GO45+). However, it increased for the large particles (GO100+). Increasing the ratio of graphite to oxidant increased the d-spacing for all the samples. The average d-spacing of GO produced from the small graphite particles (GO45) is much smaller than the other two, which have similar values.

The crystallite size is the largest (11.2–14.4 nm) for GO45 and the smallest for GO100+ (9.4–10.5 nm). This can be explained by the easier packing of the smaller sheets into a crystal lattice during drying. Increasing the G:KMnO_4_ ratio from 1:4 to 1:6 increased the crystallite size of GO45 and GO100+, while the opposite effect was observed for GO45+.

### 3.7. Thermogravimetric Analysis of GO

**Figure 10:**
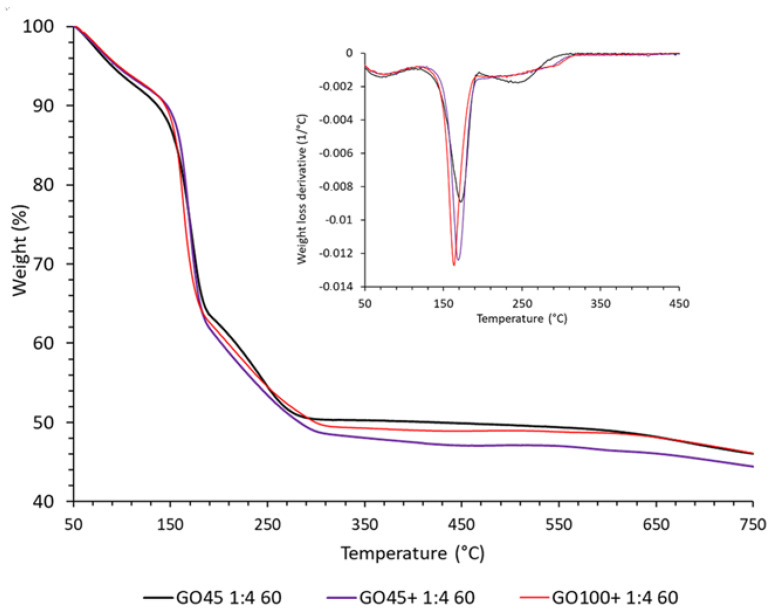
TGA curves of graphene oxides synthesized under the same conditions using graphite precursors of various sizes.

There are many reports on the TGA of GO, but only a few mention that GO can decompose explosively at a high heating rate [38,39]. In our initial experiments, explosive decomposition was observed at a heating rate of 10 °C/min and a sample mass of 10 mg. The explosion is accompanied by the deposition of fine black powder throughout the interior of the TGA instrument. In the TGA thermogram, the explosion can be recognized as an immediate loss of weight, followed by a slight increase in weight (inset in Figure 10) due to the settling of the powder back into the crucible. Sometimes the explosion is not recognized as such, and therefore incorrect mass loss data has been published [40].

To avoid an explosion during TGA characterization, the heating rate was reduced to 5 °C/min and the sample mass to about 4 mg.

The weight loss of GO, during heating, proceeds through four not well-separated steps, although the fourth step is minor and does not end at a temperature as high as 800 °C. The decomposition curves are quite similar, and therefore only three are shown as an example in Figure 10. The results of the weight loss and peak decomposition rate temperature during heating GO in the nitrogen atmosphere are summarized in Table 7. An initial mass loss (at temperatures up to about 120 °C) is usually attributed to the release of absorbed water, but could partially overlap with the onset of the decomposition at the higher temperatures. During the second step, rapid decomposition is observed, and the weight is reduced by approximately 30%. The maximum decomposition rate is observed in a temperature range of 159–173 °C.

Since no correlation between the peak decomposition temperature and the structure of GO could be derived, a TGA analysis was performed on a large batch of an older sample, synthesized from graphite of 130 μm (GO130), which was stored as a methanol slurry in a refrigerator. Part of the methanol slurry was dried first in the air and then in a vacuum at 40 °C. Another part was further purified using water, as explained in the Determination of Reaction Yield section. Dense and hard material was obtained after drying in the air. A small piece (4.5 mg) of this sample was first cut and analyzed by TGA. Another piece was crushed into smaller particles or powder using agate mortar prior to analysis. The samples of GO130, which were additionally purified by water and dried by lyophilization, had the form of thin-walled foam. The TGA was performed using both the as-synthesized and purified samples.

**Figure 11:**
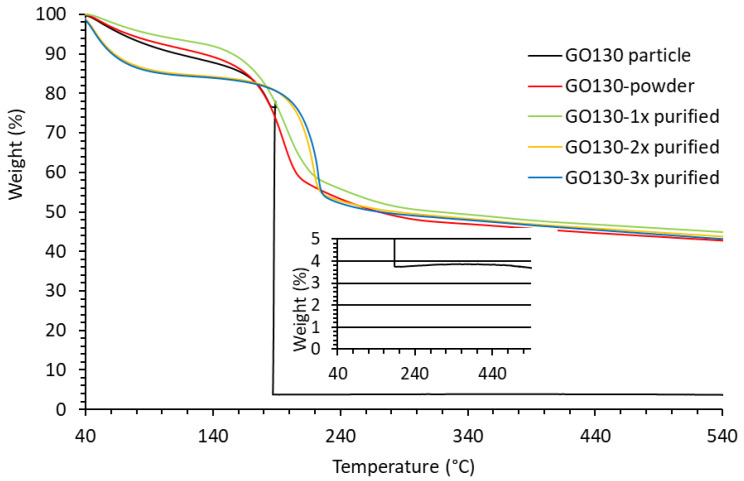
Weight loss of GO synthesized from graphite with 130 mm particle size, dried in the air (particle), crushed into powder, purified using water (1–3 times), and dried by lyophilization. Inset: Slight increase in weight after the explosive decomposition.

**Figure 12:**
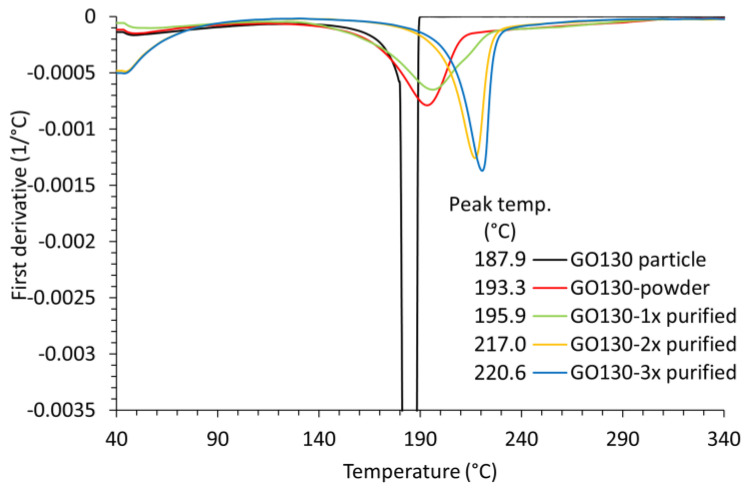
The first derivative or decomposition rate of GO130 degradation.

The thermal properties differ a lot from the other samples described in this article. The TGA thermograms and their first derivatives, which represent the decomposition rate of the GO130 samples, are shown in Figure 11 and Figure 12, respectively. The important differences were observed in the first and second decomposition steps, while the end (above 230 °C) is practically the same.

From the first step of decomposition (up to 140 °C), we can conclude that not only water evaporation but also thermal decomposition takes place. If only water is released in the first step, the mass loss rate would be faster for the powdered sample due to a larger specific surface area. However, we see that the decomposition of the powdered sample is slightly slower in the first step. The same is observed for the 1× purified sample. However, the weight loss in the first step is significantly faster for the 2× and 3× purified samples. At 100 °C, 15% of the mass was already lost. As all the purified samples were dried at the same time, we assume that this is not due to the higher moisture content in these two samples but to faster decomposition.

Large differences were also observed in the second decomposition step. The single piece exploded under the heating rate of 5 °C/min at 187.9 °C, while fast but steady decomposition was observed for the powdered sample, and the maximum decomposition rate was observed at 193 °C (the signal for the decomposition rate of the large sample in Figure 11 is large and has, therefore, been cut for clarity reasons). From this, we can conclude that the particle size of the dried GO (or rather GrO) strongly influences decomposition kinetics. The decomposition reaction is fast and exothermic. The released heat can not be dissipated from the large particle fast enough to maintain the programmed temperature. The sample is overheated, and the increased temperature of the sample further increases the rate of decomposition until it actually explodes. The overheating can be easily recognized as a loop on the heat flow–temperature diagram measured using the DSC sensor (Figure S2 in the Supplementary Materials). The smaller the particles, the easier the heat is dissipated, the lower the reaction rate, and the peak shifts to a higher temperature.

Heat dissipation is better, and the peak of decomposition shifts to a higher temperature with each purification step, as can be seen in Figure 12. The concentration of GO in water before lyophilization was 1.5%, 1.2%, and 1.0% after the first, second, and third purification steps, respectively. Therefore, the wall thickness of the GO foam decreased with each purification step, which could lead to decomposition at higher temperatures, as explained above. However, each purification step could also remove a part of the product or impurities that could catalyze decomposition. Therefore, part of GO130-3x purified was dissolved in water again, and the second part was immersed in methanol. Dry GO no longer dissolves in methanol, and even swelling is not visually recognizable. The sample remained in a powdery form. After drying in the air and in a vacuum, a TGA was performed on both samples. The peak decomposition temperature of the samples prepared in this way decreased from 220.6 °C to 218.1 °C (methanol) and from 200.5 °C to 212.4 °C for water-dissolved samples of different thicknesses. Since the peak decomposition temperature of the water-dissolved sample was higher than the temperature of GO130-1x purified, despite the thicker film, we can conclude that the decomposition kinetics depend on both the purity and the particle size of the measured sample. The slightly lower decomposition temperature observed for the GO130-3x from methanol could be due to a small solubility and an increase in particle size as an effect of methanol.

With the increasing development of new devices and technologies based on graphene [41,42,43], the need for new, high-yield methods for the synthesis of graphene is great nowadays. Table 8 summarizes the methods and GO properties. It is noticeable that the produced GO flakes show defects at a similar level as in other studies, while the flake size is in the same range, indicating that the selected procedure is appropriate for the preparation of large GO flakes with mild defects.

## 4. Conclusions

The goal of the present work was to determine the influence of graphite particle size, the ratio of graphite to oxidant (KMnO_4_), and the ratio of graphite to acid (H_2_SO_4_ and H_3_PO_4_) on the structure and properties of the synthesized GO. In this way, we wanted to find the explanation for some large deviations in the results observed in the literature.

The results reported in this paper show that these three parameters can hardly be separated due to the variations in the viscosity of the system. During oxidation, the graphite flakes became more hydrophilic, which led to swelling in the acid and increased the viscosity. The larger the graphite particles, the more viscous the paste. Due to the high viscosity, the particles can be broken by mixing, which affects their size. This could be an important reason why the size of the GO particles made from large graphite flakes is not much larger than the size of the GO particles made from smaller graphite flakes. However, the results show that the particle size of the dried GO is 50% larger than before drying. This result can be explained by the formation of a chemical bond between neighboring GO sheets during drying, which prevents complete exfoliation in water or even swelling in methanol.

According to the literature sources, the reaction yield varies a lot. Our results show that the main reason for this is the purification method. The more water is used, the lower the GO yield. Interestingly enough, when we compared the amount of sulfur in our GO samples with the others that used more rigorous purification and more water, quite similar values were obtained. This means that excessive purification leads to the excessive waste of good and valuable GO and not necessarily to a purer GO.

Increasing the ratio of graphite to KMnO_4_ from 1:4 to 1:6 generally led to an increase in the degree of oxidation and the reaction yield. However, the differences are very small. Increasing the amount of acids from 60 mL to 80 mL led to a lower degree of oxidation and a slightly lower reaction yield, except for the smallest particles.

The measurement of the water contact angle showed no correlation between the wettability and the degree of oxidation. The highest water contact angle of 34.3° was measured for the GO100+ 1:4:80 sample, which had the lowest O/C ratio. The variations in the contact angles of the other samples could be related to the lack of uniformity of the films, which was influenced by the deposition method or by variations in the oxidation state. Epoxy and C=O groups contribute to oxidation but are not as polar as hydroxy and -COOH. Unfortunately, XPS analysis cannot distinguish between them, so the actual structure cannot be deduced from high-resolution XPS C1s spectra.

The crystallite size of GO is largest (11.2–14.4 nm) for the smallest particles (GO45) and smallest (9.4–10.5 nm) for the largest particles (GO100+). This can be explained by the fact that the smaller sheets fit more easily into a crystal lattice during drying. It decreases with increasing acidity (dilution) and increases with the increasing ratio of graphite to the oxidizing agent from 1:4 to 1:6.

The thermal decomposition of GO proceeds in four not-well-separated steps. The second step is the fastest and can even be explosive, especially when large samples and/or high heating rates are used. The temperature of the maximum decomposition rate of this step depends not only on the synthesis and purification conditions but primarily on the size of the particles. If the GO particle is thick, the released heat cannot be dissipated fast enough to maintain the programmed temperature. The sample will overheat, and the increased temperature will further stimulate the decomposition rate until it explodes. Although the further purification of the GO (up to three times) with water increased the decomposition temperature by up to 22 °C, it was shown that the increase was mainly due to the thinner particles obtained after drying by lyophilization.

The synthesis of GO is time-consuming and ecologically unfriendly. The results presented in this article give some guidelines for optimization, especially in terms of increasing reaction yield, which might be even increased if less water and more methanol are used. The properties of GO are changing over time, which is a topic that has not been tackled in the literature. How the particle size changes over time when GO is dried or if stored in the form of a slurry (water or methanol) are interesting questions that still need to be answered. In this article, we have given some explanations for the different thermal stabilities of GO, but it would be good to investigate in more detail the influence of the composition, the presence of other elements (Cl, S, etc.), and the time that has passed since the synthesis of the samples on the thermal stability of GO.

## Figures and Tables

**Table 1:** The yield of GO, calculated as a weight of GO divided by the weight of graphite used in the reaction, depending on the graphite particle size (<45 μm, 45–100 μm, and >100 μm), graphite-to-KMnO_4_ ratio (1:4 or 1:6), and quantity of acids (60 mL or 80 mL).

	Yield (%)		Yield (%)		Yield (%)
GO45 1:4:60	210	GO45+ 1:4:60	223	GO100+ 1:4:60	223
GO45 1:4:80	217	GO45+ 1:4:80	218	GO100+ 1:4:80	223
GO45 1:6:60	226	GO45+ 1:6:60	226	GO100+ 1:6:60	227
GO45 1:6:80	228	GO45+ 1:6:80	222	GO100+ 1:6:80	226

**Table 2:** The proportions of various carbon bonds together with their oxygen-to-carbon atomic ratios.

	C-C/C=C (%)	C-O (%)	C=O (%)	COOH (%)	O/C
GO45 1:4:60	43.7	44.7	8.1	3.5	0.52
GO45 1:4:80	38.7	47.4	9.8	4.1	0.58
GO45 1:6:60	35.5	50.2	10.4	3.8	0.62
GO45 1:6:80	38.2	45.1	11.9	4.8	0.58
GO100+ 1:4:60	43.6	44.0	7.9	4.5	0.55
GO100+ 1:4:80	47.8	45.0	3.5	3.7	0.44
GO100+ 1:6:60	42.5	46.1	7.4	4.0	0.54
GO100+ 1:6:80	44.7	45.8	5.9	3.6	0.50

**Table 3:** *I_D_*/*I_G_* ratio values of the synthesized GO samples.

Sample	*I_D_/I_G_* Ratio	Sample	*I_D_/I_G_* Ratio
GO45 1:4:60	0.92	GO100+ 1:4:60	0.89
GO45 1:4:80	0.92	GO100+ 1:4:80	0.92
GO45 1:6:60	0.95	GO100+ 1:6:60	0.92
GO45 1:6:80	0.92	GO100+ 1:6:80	0.88

**Table 4:** Water contact angle measurements of the as-synthesized GO samples.

Sample	Contact Angle	Sample	Contact Angle
GO45 1:4:60	21.0°	GO100+ 1:4:60	24.0°
GO45 1:4:80	22.6°	GO100+ 1:4:80	34.3°
GO45 1:6:60	28.5°	GO100+ 1:6:60	25.3°
GO45 1:6:80	28.3°	GO100+ 1:6:80	23.8°

**Table 5:** GO particle size measured using DLS in demineralized water from the GO slurry in methanol and dried GO samples.

	Slurry		Dried Samples	
	Z-aver. (nm)	PDI	Z-aver. (nm)	PDI
GO45 1:4:60	5249	0.524	7020	0.730
GO45 1:4:80	3984	0.407	5062	0.569
GO45 1:6:60	2637	0.416	4579	0.834
GO45 1:6:80	2851	0.364	4868	0.784
GO45+ 1:4:60	2800	0.493	3688	0.573
GO45+ 1:4:80	2577	0.530	4161	0.535
GO45+ 1:6:60	3418	0.682	4465	0.892
GO45+ 1:6:80	3701	0.528	5438	0.454
GO100+ 1:4:60	3486	0.253	5420	0.406
GO100+ 1:4:80	4096	0.368	7131	0.568
GO100+ 1:6:60	5151	0.639	7187	0.885
GO100+ 1:6:80	4306	0.472	6141	0.508

**Table 6:** The peak position, d-spacing, full width at half maximum (FWHM), and crystallite size of the synthesized graphene oxides.

Sample	2θ (°)	d-Spacing (nm)	FWHM	Cryst. Size (nm)
GO45 1:4:60	9.765	0.905	0.659	13.3
GO45 1:4:80	9.907	0.892	0.862	9.9
GO45 1:6:60	9.661	0.915	0.613	14.4
GO45 1:6:80	9.731	0.908	0.774	11.1
GO45+ 1:4:60	9.521	0.928	0.858	10.0
GO45+ 1:4:80	9.543	0.926	0.751	11.5
GO45+ 1:6:60	9.357	0.944	0.752	11.5
GO45+ 1:6:80	9.486	0.932	0.687	12.7
GO100+ 1:4:60	9.754	0.906	0.834	10.3
GO100+ 1:4:80	9.455	0.935	0.910	9.4
GO100+ 1:6:60	9.638	0.917	0.819	10.5
GO100+ 1:6:80	9.318	0.948	0.908	9.4

**Table 7:** Weight loss and peak decomposition rate temperature during heating GO in a nitrogen atmosphere were determined by TGA.

	Weight loss				Peak
	1st st. (%)	2nd st. (%)	3rd st. (%)	4th st. (%)	(°C)
GO45 1:4:60	8.8	28.9	11.7	6.2	172
GO45 1:4:80	8.5	30.3	12.7	4.6	162
GO45 1:6:60	8.1	32.7	13.5	3.4	170
GO45 1:6:80	7.9	29.8	12.8	4.0	159
GO45+ 1:4:60	7.7	31.8	13.4	4.4	168
GO45+ 1:4:80	9.3	30.9	13.5	6.0	173
GO45+ 1:6:60	7.7	31.3	12.0	4.7	170
GO45+ 1:6:80	7.8	29.9	11.6	3.8	173
GO100+ 1:4:60	7.4	30.0	13.1	6.5	163
GO100+ 1:4:80	8.0	30.4	13.6	3.0	164
GO100+ 1:6:60	7.5	31.3	12.0	3.7	170
GO100+ 1:6:80	7.8	31.1	14.5	4.4	166

**Table 8:** Comparison of GO properties produced in different synthetic procedures.

Method	*I_D_/I_G_*	Sheet Size (μm)	TGA WL 500 °C (%)	XRD (°)	Ref.
Electrochemical followed by oxidation with KMnO_4_	1.24	≈10	≈80	8.88	[44]
Hummers’ method	0.66–0.90	0.98	61.55	10.50	[45]
Improved Hummers’ method with boric acid	0.92–1.04	/	46.22	10.10	[46]
Modified Hummers’	1.0	≈10	/	/	[47]
Modified Hummers’	0.83	/	/	10.44	[48]
Tour method	1.77	/	/	10.44	[49]
Electrochemical synthesis	0.41–1.71	/	/	9–12	[50]
Electrochemical synthesis	0.04	Up to 12			[51]
Modified Hummers’	0.92	Up to 5.25	/	9.91	This study

## Data Availability

Data obtained within the Grinshield project will be published on Zenodo after the article is accepted for publication.

## Supplementary Materials

The following supporting information can be downloaded at: https://www.mdpi.com/article/10.3390/nano14030281/s1, Figure S1: Individual high-resolution C1s spectra of the investigated samples with subcomponents positioned at 284.8, 287.0, 288.0, and 289.0 eV. Figure S2: Heat flow signal of TGA-DSC measurement.
