# Supplementary material for: The Influence of Reaction Conditions on the Properties of Graphene Oxide
Source: Nanomaterials (Basel). 2024 Jan 30;14(3):281. doi: 10.3390/nano14030281 (PMC10856647; doi:10.3390/nano14030281)
Supplement: Supplementary file 1 [file nanomaterials-14-00281-s001.zip › nanomaterials-2807665-supplementary.pdf]

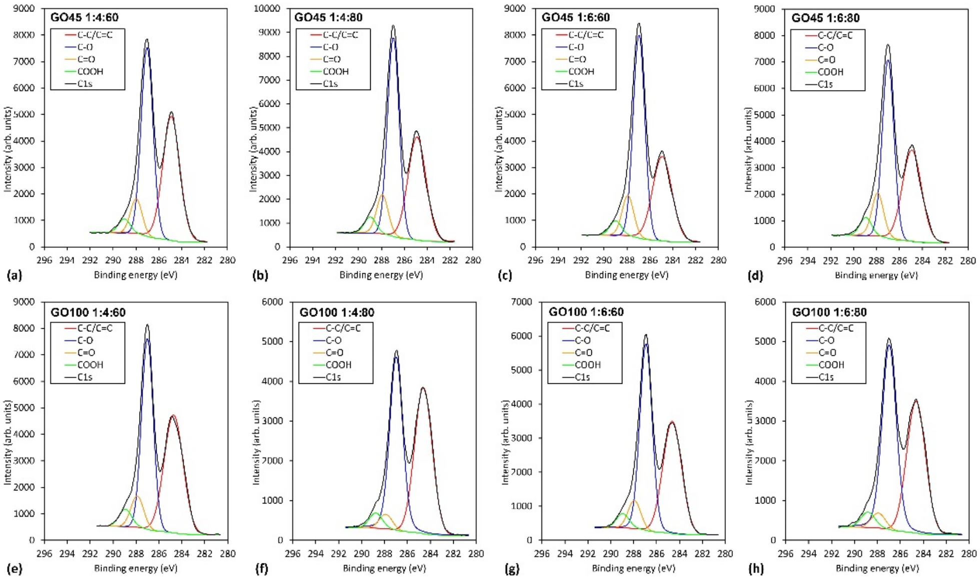

Figure S1: Individual high-resolution C1s spectra of the investigated samples with subcomponents positioned at 284.8, 287.0, 288.0, 289.0 eV.

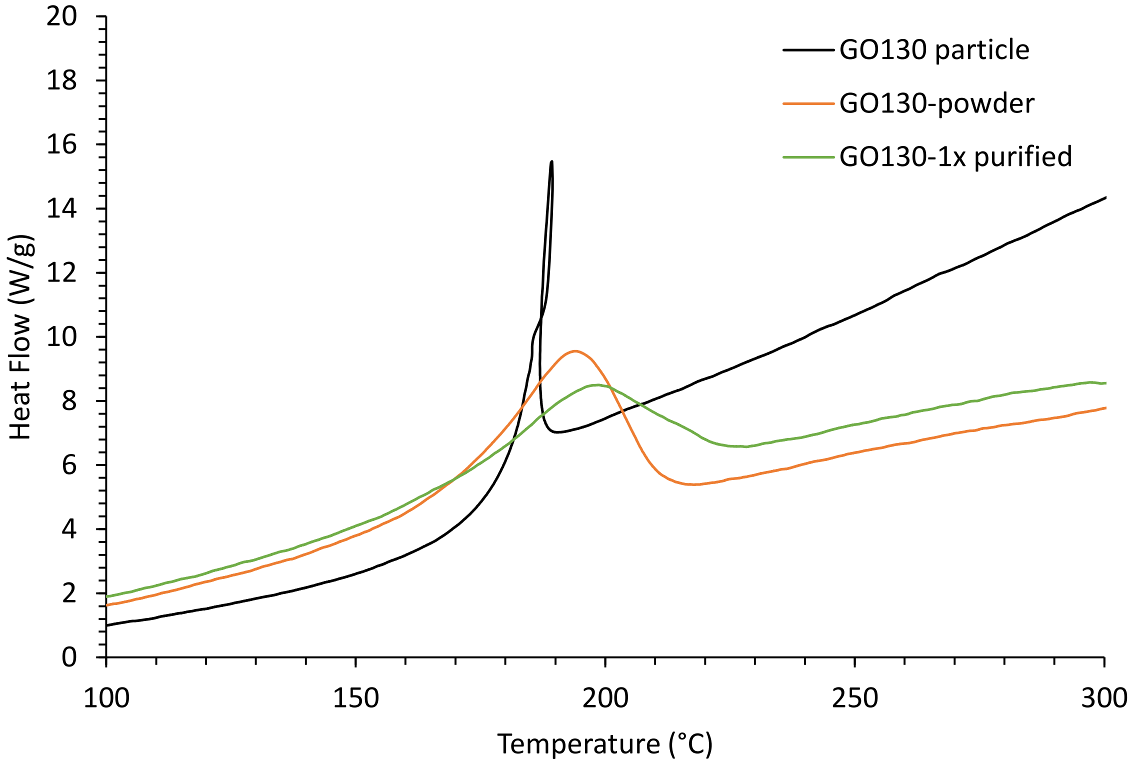

Figure S2: Heat flow signal of TGA-DSC measurement.
